# Co-evolution of cancer microenvironment reveals distinctive patterns of gastric cancer invasion: laboratory evidence and clinical significance

**DOI:** 10.1186/1479-5876-8-101

**Published:** 2010-10-15

**Authors:** Chun-Wei Peng, Xiu-Li Liu, Xiong Liu, Yan Li

**Affiliations:** 1 Department of Oncology, Zhongnan Hospital of Wuhan University, Hubei Key Laboratory of Tumor Biological Behaviors & Hubei Cancer Clinical Study Center, Wuhan 430071, China

## Abstract

**Background:**

Cancer invasion results from constant interactions between cancer cells and their microenvironment. Major components of the cancer microenvironment are stromal cells, infiltrating inflammatory cells, collagens, matrix metalloproteinases (MMP) and newly formed blood vessels. This study was to determine the roles of MMP-9, MMP-2, type IV collagen, infiltrating macrophages and tumor microvessels in gastric cancer (GC) invasion and their clinico-pathological significance.

**Methods:**

Paraffin-embedded tissue sections from 37 GC patients were studied by Streptavidin-Peroxidase (SP) immunohistochemical technique to determine the levels of MMP-2, MMP-9, type IV collagen, macrophages infiltration and microvessel density (MVD). Different invasion patterns were delineated and their correlation with major clinico-pathological information was explored.

**Results:**

MMP2 expression was higher in malignant gland compared to normal gland, especially nearby the basement membrane (BM). High densities of macrophages at the interface of cancer nests and stroma were found where BM integrity was destroyed. MMP2 expression was significantly increased in cases with recurrence and distant metastasis (*P = *0.047 and 0.048, respectively). Infiltrating macrophages were correlated with serosa invasion (*P *= 0.011) and TNM stage (*P *= 0.001). MVD was higher in type IV collagen negative group compared to type IV collagen positive group (*P *= 0.026). MVD was related to infiltrating macrophages density (*P *= 0.040). Patients with negative MMP9 expression had better overall survival (OS) compared to those with positive MMP9 expression (Median OS 44.0 vs 13.5 mo, *P *= 0.036). Median OS was significantly longer in type IV collagen positive group than negative group (Median OS 25.5 vs 10.0 mo, *P *= 0.044). The cumulative OS rate was higher in low macrophages density group than in high macrophages density group (median OS 40.5 vs 13.0 mo, *P *= 0.056). Median OS was significantly longer in low MVD group than high MVD group (median OS 39.0 vs 8.5 mo, *P *= 0.001). The difference of disease-free survival (DFS) between low MVD group and high MVD group was not statistically significant (*P *= 0.260). Four typical patterns of cancer invasion were identified based on histological study of the cancer tissue, including Washing pattern, Ameba-like pattern, Spindle pattern and Linear pattern.

**Conclusions:**

Proteolytic enzymes MMP9, MMP2 and macrophages in stroma contribute to GC progression by facilitating the angiogenesis. Cancer invasion patterns may help predict GC metastasis.

## Background

Tumor progression represents the greatest threat to patients with gastric cancer (GC). The 5-year survival is significantly decreased from over 80% in early GC to below 28% in advanced GC [1]. Over the past 25 years, the majority of cancer studies have focused on functional consequences of activating and/or inactivating mutations in critical genes and signal pathways that regulate cell proliferation and/or cell death as cancer is often defined as a disease of cell proliferation [2]. However, such studies have largely ignored the fact that interactions between cancer cells and stroma are critical for growth and invasion of epithelial tumors [3]. It has been recognized that invasion is regulated not only by intrinsic genetic changes in cancer cells as 'initiators' of carcinogenesis, but also regulated by stroma cell as 'promoter' [4,5]. A seminal event in cancer progression is the ability of cancer cells to mobilize the necessary machinery to break surrounding extracellular matrix (ECM) barriers while orchestrating a host stroma response that ultimately supports tissue-invasive and metastatic processes [6]. Proteolytic ECM remodeling is considered both prerequisite and consequence of invasive cell migration [7]. The cancer cell and stroma both modulate the process of invasion by remodeling the ECM with tumor-associated proteases such as matrix metalloproteinase (MMPs), which subsequently breakdown proteins of the ECM such as collagens and release the cryptic information [8,9]. Many studies have focused on the role of extracellular proteases. It was supposed that cancer cells break through the ECM barriers and invade surrounding tissues in two fashions: a protease-independent and Rho kinase (ROCK)-dependent amoeboid migration mode and a protease-dependent and ROCK-independent mesenchymal migration mode [10]. Further more, the process of pericellular proteolysis leads to ECM degradation and realignment during cell movement and integrate it into established steps of cell migration [11].

**Figure 1:**
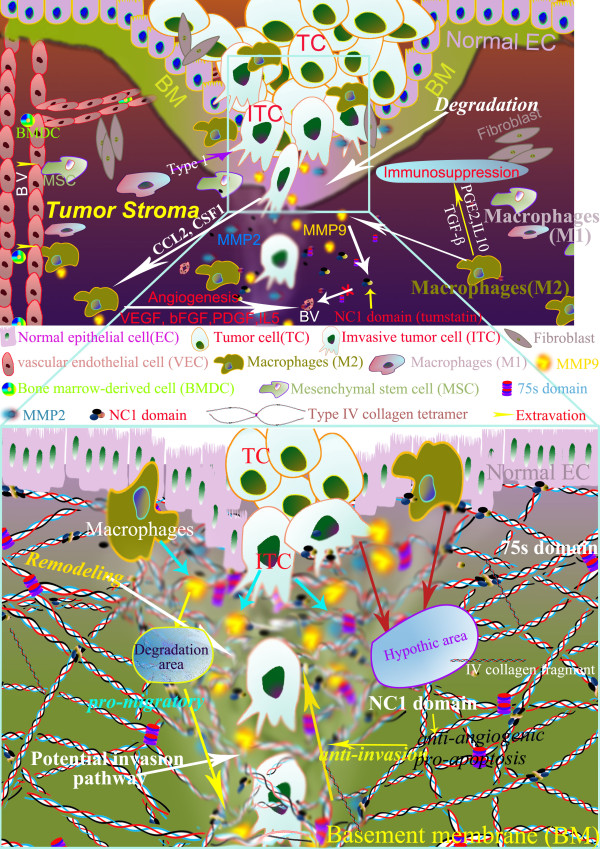
**Co-evolution of tumor cells and their microenvironment in cancer invasions**. Both of tumor cells and their microenvironment are involved in cancer invasions. Invasion is the first observable step of cancer progression process that tumor cells cross the ECM barrier by proteolytic enzyme such as MMPs after acquiring invasive phenotypes (upper graph). In addition, tumor infiltrating macrophages and type IV collagen also play an important role in cancer invasion. In this process, cancer invasion networks capture "temporal evolution" and "spatial evolution" between tumor cells and microenvironment before mechanical macrotrack can be observed as stroma remodelling at the histological level (lower graph).

It has long been recognized that the behavior of tumor systems is complex, which means that understanding the individual component like pericellular proteolysis in more detail does not necessarily explain the collective behavior of many individuals, and thus usually evokes Aristotle's quote in that 'The whole is more than the sum of its parts' [12]. Therefore, instead of investigating a single component of cancer matrix, this study focused on the whole tumor microenvironment related to GC invasion, by evaluating tissue destructive proteolytic enzymes MMP9 and MMP2, tissue barriers against invasion like type IV collagen, tumor infiltrating macrophages, and tumor angiogenesis, all of which are essential components of tumor stroma and involved in the process of invasion (Figure 1.). Furthermore, the interactions between cancer cells and tumor stroma termed as 'invasion pattern' corresponding to the dynamic stroma remodeling were also delineated so as to formulate new concepts on cancer invasion at the histological level.

## Methods

### Patients and tissue samples

Tumor specimens were obtained from 37 GC patients at the Department of Oncology, Zhongnan Hospital of Wuhan University (Wuhan, China) from January 2004 to January 2008. Written informed consent was obtained from the patients and the study protocol was approved by the ethics committee of Zhongnan Hospital of Wuhan University. Major clinico-pathological features of these patients were listed in Table 1. The patients underwent curative gastrectomy with D2 lymph nodes dissection for stages I to III cases and palliative surgery for some stage IV cases. Tumor staging was based on TNM classification system of American Joint Committee on Cancer (AJCC) staging criteria (version 6). All patients beyond stage II received platinum and 5-flurouracil (5-FU) based adjuvant chemotherapy beginning 21 days after surgery. The last follow-up was on December 1, 2009.

**Table 1:** Clinicopathological characteristics in relation to MMP9, MMP2, Type IV collagen and Macrophages immunoreactivity

Variables	N	MMP9 Positive (%)	P*	MMP2 positive (%)	P*	Type IV collagen Positive (%)	P*	Macrophages counts (M ± SD)	P**
Age (yr)									
≤ 58	18	13 (72.2)	NS	9 (50.0)	NS	12 (66.7)	**0.042**	19.9 ± 10.6	NS
> 58	19	17 (89.5)		11 (57.9)		18 (94.7)		19.4 ± 7.3	
Recurrence									
No	13	10 (76.9)	NS	4 (30.8)	**0.047^#^**	10 (76.9)	NS	17.3 ± 7.9	NS
Yes	24	20 (83.3)		16 (66.7)		20 (83.3)		21.0 ± 9.4	
Serosa invasion									
No	8	7 (87.5)	NS	4 (50)	NS	7 (87.5)	NS	12.7 ± 9.2	**0.011**
Yes	29	23 (79.3)		16 (55.2)		23 (79.3)		21.6 ± 8.0	
Lymph node metastasis									
No	10	8 (80.0)	NS	3 (30.0)	NS	10 (100)	NS	16.3 ± 8.3	NS
Yes	27	22 (81.5)		17 (63.0)		20 (74.1)		20.9 ± 9.0	
Distant Metastasis									
M0	29	23 (79.3)	NS	13 (44.8)	**0.048**	25 (86.2)	NS	18.9 ± 8.3	**0.09**
M1	8	7 (87.5)		7 (87.5)		5 (62.5)		22.6 ± 11.0	
TNM Stage									
Early	11	9 (81.8)	NS	3 (27.3)	NS	10 (90.9)	NS	12.8 ± 7.1	**0.001**
Advanced	26	21 (80.8)		17 (65.4)		20 (76.9)		22.6 ± 8.1	

* Fisher's exact test (two-tailed), bold face representing significant data (*P *< 0.05), NS: No statistically significant.

** t-test (two-tailed), bold face representing significant data (*P *< 0.05), NS: No statistically significant.

# The differences of MMP2 expression among different recurrence area (distant recurrence, local recurrence and ovarian recurrence) are statistically significant, too (*P *= 0.024).

### Immunohistochemistry

Immunolocalization of MMP9, MMP2, type IV Collagen, macrophages and CD105 were performed using streptavidin-biotin peroxidase complex method (SP). Briefly, tissue slides were first deparaffinized in xylene, ethanol and water, then the slides were pretreated in 0.01 M citrate buffer (pH 6.0) for MMP9, MMP2, macrophages or 1 mM EDTA (pH 9.0) for CD105, and heated in a microwave oven (98°C) for 10 min. For staining, endogenous peroxidase activity was blocked by immersing in 3% H_2_O_2 _in methanol for 10 min to prevent any nonspecific binding. After blocked with 2% BSA, the slides were incubated with the primary antibodies for MMP9 (sc13595, Santa Cruz, USA, dilution 1/300), MMP2 (sc-6840, Santa Cruz, USA, dilution 1/300), type IV collagen (ab6586, Abcam, England, dilution 1/300), macrophages (MA1-38069, ABR, USA, dilution 1/300), and CD105 (sc-23838, Santa Cruz, USA, dilution 1/300) for 90 min at 37°C, then incubated with the corresponding secondary antibody for 15 min at 37°C, and finally incubated with peroxidase-labeled streptavidin (Maixin Biotechnology, China) for 15 min. The reaction products were visualized with diaminobenzidine (DAKO, Denmark). All slides were counterstained with haematoxylin. As a negative control, primary antibody was replaced with Tris-buffered saline on sections that were proven to be positive for MMP9, MMP2, type IV collagen, macrophages and CD105 in preliminary experiments.

### Evaluation of Immunohistochemical Variables

Positive cells were stained brownish granules. The infiltrating macrophages were counted in five high power fields selected at the tumor invasion front, and the mean cells counts were documented. Because CD105 is a specific marker of newly formed and activated small blood vessels, the MVD was calculated as the average count from the three hotspot fields of view and used for analysis of angiogenesis. The percentage of immunoreactive positive cells and intensity for MMP9, MMP2, type IV collagen in GC were assessed. All slides were independently observed by two investigators. The staining score of each slide was calculated by staining intensity and percentage of positive cancer cells. The staining intensity was scored as 1 (very weak), 2 (weak), 3 (moderate), 4 (intense) and 5 (very intense). Positive rate score of cancer cells was: 0-10% was recorded as 0; 10-30% was recorded as 1; 30-50% was recorded as 2; 50-75% was recorded as 3; > 75% were recorded as 4. The expression of MMP9, MMP2 and type IV collagen, and macrophages infiltration in each slide were scored as the sum of intensity and positive rate scores. Negative was defined as the score ≤ 3 for MMP9, MMP2 and type IV collagen.

### Statistical Analysis

Statistical analyses were performed with SPSS software version 13.0 (SPSS Inc. Chicago, IL). Cumulative survival was calculated by the Kaplan-Meier method and analyzed by the Log-rank test. A secondary analysis was performed to assess the relationship among immunohistochemical variables and clinicopathological characteristics. For the comparison of individual variables, Fisher's exact test, t test and Mann-Whitney Test were conducted as appropriate. Two-tailed *P *< 0.05 was judged to be significant.

## Results

### Immunohistochemical characteristics

**Figure 2:**
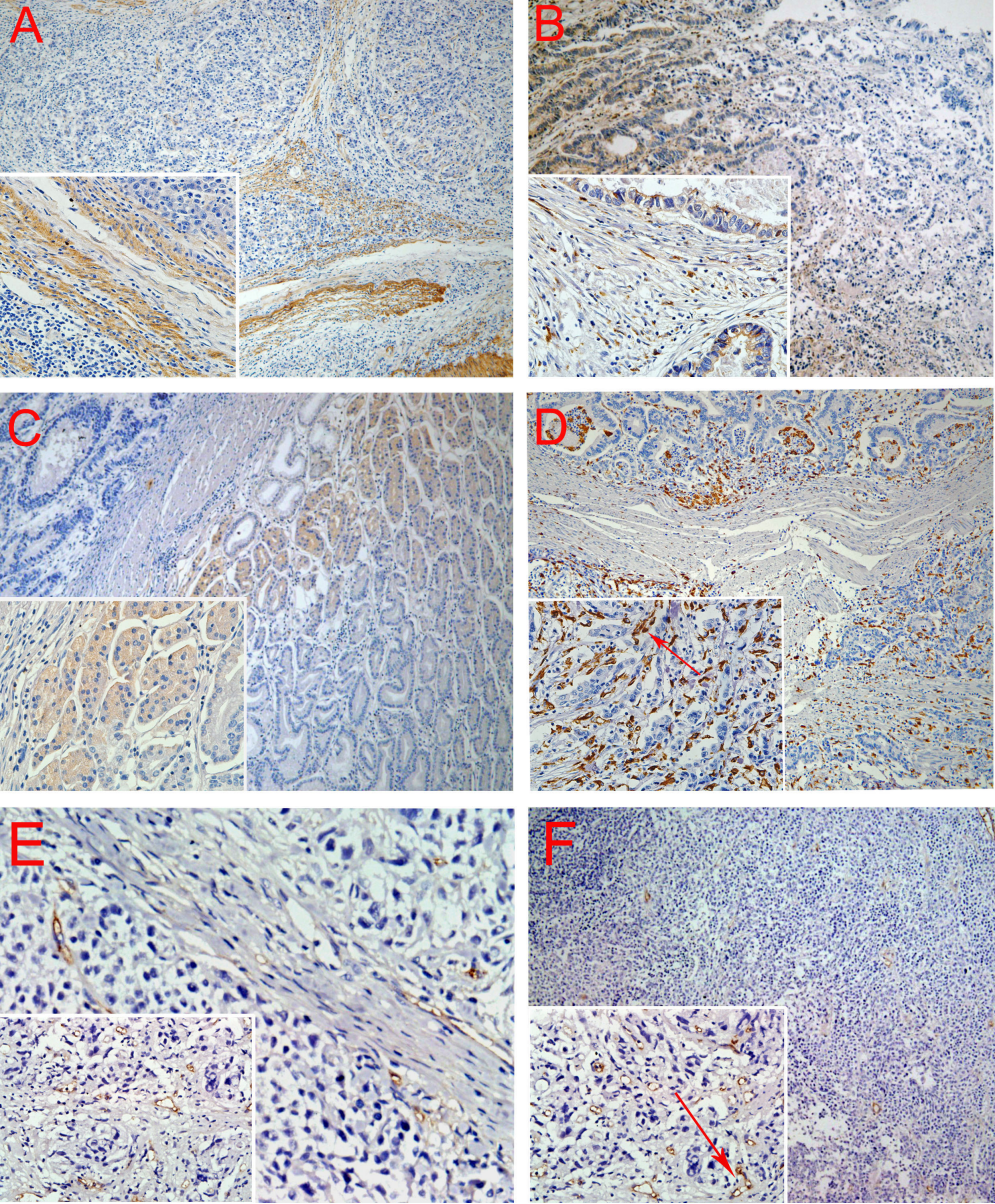
**Positive staining of type IV collagen, MMP9, MMP2, macrophages, and microvessels**. A. BM was revealed by type IV Collagen staining. B. MMP9 was secreted by GC cells and mesenchymal. C. MMP2 expression is higher in malignant gland versus normal gland, especially nearby the BM. D. Macrophages are mainly located in the margin of the tumor nest, and phagocytosis of cancer cells by macrophage was observed (red arrow). E. New microvessels were increased at the tumor front. And CD105 is highly expressed on proliferating endothelial cells of both the peri- and intratumoral blood vessels (red arrow). Magnifications: A, B, C, D, E, F: 100×; Inserts in lower left corner show the sub-cellular localization of immunostaining at higher magnification (400×). All tissues were adenocarcinoma of GC.

Immunohistochemical analysis showed the linearity of type IV collagen was disrupted indicating BM destruction (Figure 2A). The characteristic distribution pattern of MMP9 was diffused expression in tumor tissue, although small areas of scattered expression were also observed (Figure 2B). Furthermore, MMP2 expression was higher in malignant gland compared to normal gland, especially nearby the BM (Figure 2C). High density of macrophages was observed at the juncture of cancer cells and stroma where BM integrity of gastric gland had been broken (Figure 2D). CD105 was expressed in the endothelium of blood vessels, but not in GC cells. The number of CD105-positive vessels was increased at the tumor front (Figure 2E). And CD105 is highly expressed on proliferating endothelial cells of both the peri- and intratumoral blood vessels (Figure 2F).

### Correlation of Immunohistochemical Variables with clinicopathologic features

Serosa invasion, lymph node status, TNM stages, recurrence status and distant metastasis were the variables investigated in this study, all of which were not related to the level of MMP9 and IV collagen, but IV collagen expression was significantly decreased in older patients (*P *= 0.042). MMP2 expressions were significantly increased in cases with recurrence and distant metastasis (*P *= 0.047 and 0.048, respectively). Moreover, the expression of MMP2 expression was highest in distant recurrence and lowest in local recurrence (*P *= 0.024). macrophages infiltrating level was significantly higher in cases with serosa-invasion (21.6 ± 8.0) than those without serosa-invasion (12.7 ± 9.2) (*P *= 0.011); and higher in advanced GC (22.6 ± 8.1) than early GC (12.8 ± 7.1) (*P *= 0.001). Moreover, MVD was higher in high density macrophages group than in low density group (*P *= 0.040). Lymph node metastasis and TNM stage were correlated with MVD (*P *values are 0.019 and 0.010, respectively). Especially, MVD was higher in type IV collagen negative group than in positive group (*P *= 0.026). Major information was summarized in table 1 and table 2.

**Table 2:** Analysis of tumor angiogenesis related factors

Variables	MVD		
	
	N	Median (Range)	P*
IV Collagen			
Negative	7	25 (16-30)	**0.026**
Positive	30	13 (2-33)	
Macrophages			
Low density group	16	9 (2-30)	**0.040**
High density group	21	18 (8-33)	
Serous invasion			
No	8	11 (2-30)	0.260
Yes	29	18 (5-33)	
Lymph Node metastasis			
No	10	8 (2-33)	**0.019**
Yes	27	19 (6-32)	
TNM Stage			
Early	11	9 (2-30)	**0.010**
Advanced	26	19 (6-33)	

*Mann-Whitney Test (two-tailed), bold face representing significant data (*P *< 0.05), NS: No statistically significant.

### Analysis of factors related to overall survival (OS) and Disease-Free Survival (DFS)

At the time of last follow-up, 30 patients died, 1 survived with disease and 6 survived free of disease. The median OS and median DFS were 19.0 and 10.0 months, respectively.

With regard to traditional clinico-pathological features, OS was correlated with serosa invasion, distant metastasis and TNM stages (*P *= 0.024, 0.021 and 0.009, respectively); and DFS was related to serosa invasion and TNM stages (*P *= 0.038 and 0.006, respectively). With regard to key molecular features in this study, the OS was longer in MMP9 negative group (44.0 months) than in MMP9 positive group (13.5 months) (*P *= 0.036), in type IV collagen positive group (25.5 months) than negative group (10.0 months) (*P *= 0.044), and in MMP2 negative group (22.0 months) than in MMP2 positive group (14.0 months) (*P *= 0.867), although the differences in MMP2 expression did not reach statistical significance. The OS was shorter in patients with high density of infiltrating macrophages (13.0 months) than those with in low density (40.5 months), but the significance was only marginal (*P *= 0.056). The OS was significantly shorter with High MVD than those with low MVD (*P *= 0.001).

**Figure 3:**
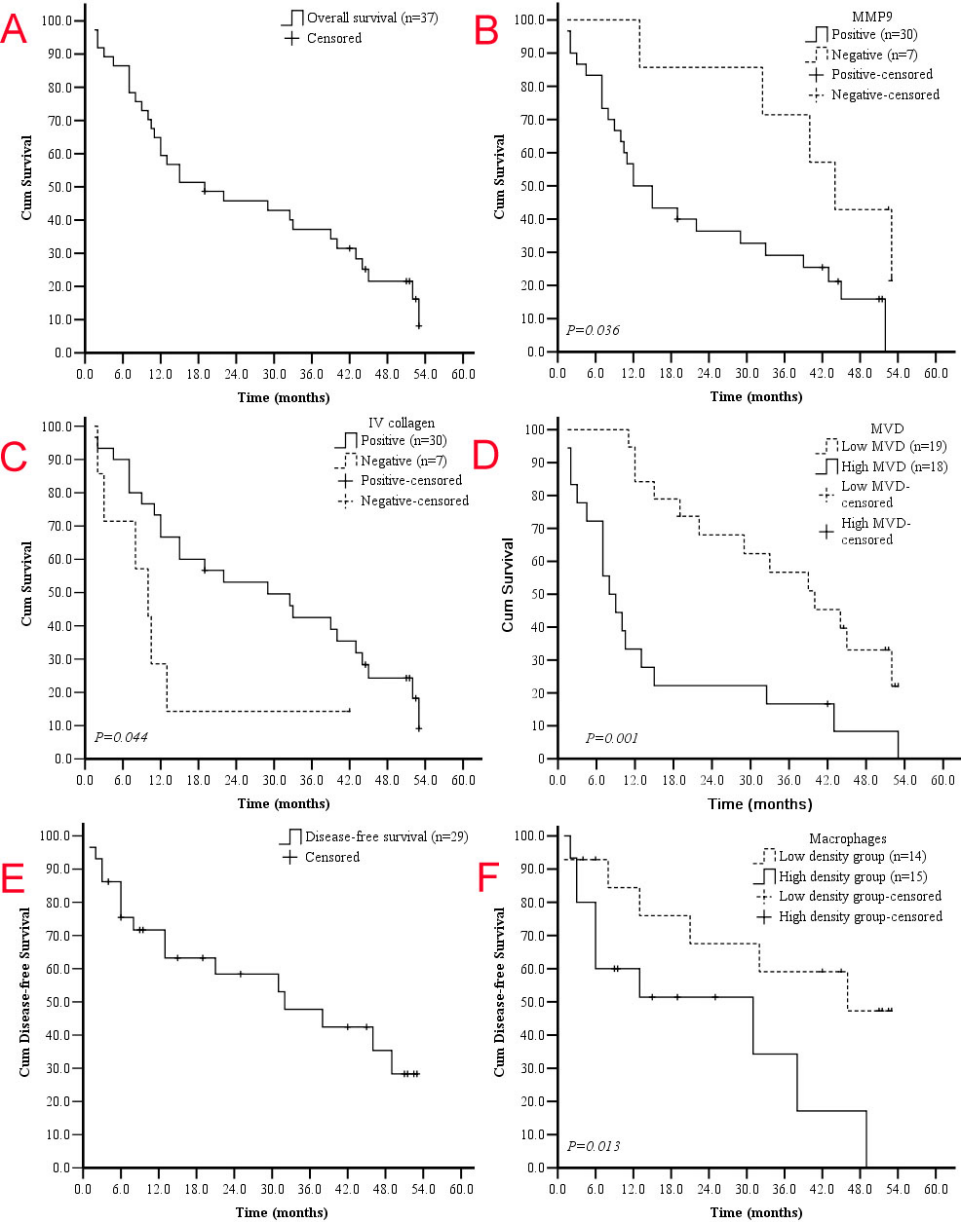
**Kaplan-Meier analysis of overall survival (OS) and disease-free survival (DFS)**. The median OS and DFS for 37 patients overall and 29 patients without distant metastasis were 19.0 and 10.0 months, respectively (A, E). GC patients with negative MMP9 expression (-) displayed better OS (B, upper curve) compared to those with positive MMP9 expression (B, lower curve) (*P *= 0.036, Log-rank test). Type IV collagen is a protective factor for GC patients (C, *P *= 0.044, Log-rank test). High MVD may predict poor OS (D, *P *= 0.001, Log-rank test). Low density of infiltrating Macrophages showed a tendency towards favorable DFS. Patients in low density of infiltrating macrophages group expression displayed improve DFS (F, upper curve) compared to patients with high density group expression (F, lower curve) (*P *= 0.013, Log-rank test).

In terms of DFS, the study did not reveal any correlation between DFS with expression levels of MMP9, MMP2, type IV collagen, or MVD. In contrast, DFS was longer in low macrophages density group (37.0 months) than in high density group (9.5 months) (P = 0.013). Key results were summarized in Table 3 and Figure 3.

**Table 3:** The analyses of factors regarding OS and disease-free survival

Variables	OS			DFS		
		
	N	Median (Range)	P*	N	Median (Range)	P*
Clinico-pathological data						
Pathological types						
Adenocarcinoma	25	19.0 (1.5-52.5)	0.860	20	14.0 (1.0-52.5)	0.796
Nonadenocarcinoma	12	22.0 (3.0-53.0)		9	25.0 (2.0-53.0)	
Serosa invasion						
No (T1/T2)	8	44.0 (2.0-52.5)	**0.024**	7	45.0 (13.0-52.5)	**0.038**
Yes (T3/T4)	29	13.0 (1.5-53.0)		22	9.3 (1.0-53.0)	
Lymph node metastasis						
No	10	22.0 (1.5-53.0)	0.213	9	38.0 (6.0-52.5)	0.681
Yes	27	12.5 (2.0-33.0)		20	11.3 (1.0-53.0)	
Distant metastasis						
M0	29	22.0 (1.5-53.0)	**0.021**			
M1	8	12.5 (2.0-33.0)				
TNM stage						
Early	11	44.0 (11.0-52.5)	**0.009**	11	46.0 (42.0-52.5)	**0.006**
Advanced	26	12.0 (1.5-53.0)			18	26.5 (8.0-51.5)
Immunohistochemistry (IHC)						
MMP9						
Positive	30	13.5 (1.5-52.0)	***0.036***	23	9.5 (1.0-51.5)	0.171
Negative	7	44.0 (13.0-53.0)		6	43.5 (25.0-53.0)	
MMP2						
Positive	20	14.0 (1.5-53.0)	*0.867*	13	9.0 (1.0-53.0)	0.395
Negative	17	22.0 (7.0-51.5)		16	20.0 (4.0-51.5)	
Type IV collagen						
Positive	30	25.5 (1.5-53.0)	***0.044***	25	19.0 (1.0-53.0)	0.646
Negative	7	10.0 (2.0-42.0)		4	9.3 (2.0-42.0)	
Macrophages						
Low density	16	40.5 (1.5-53.0)	0.056	16	37.0 (1.0-53.0)	**0.013**
High density	21	13.0 (2.0-53.0)		21	9.5 (2.0-49.0)	
MVD						
Low	19	39.0 (11.0-53.0)	0.001	16	21 (3.0-53)	0.209
High	18	8.5 (1.5-53.0)		13	6.0 (0.5-49.0)	

* Log-rank test (Two-tailed), bold font representing significant data (*P *< 0.05).

### Patterns of invasion

**Figure 4:**
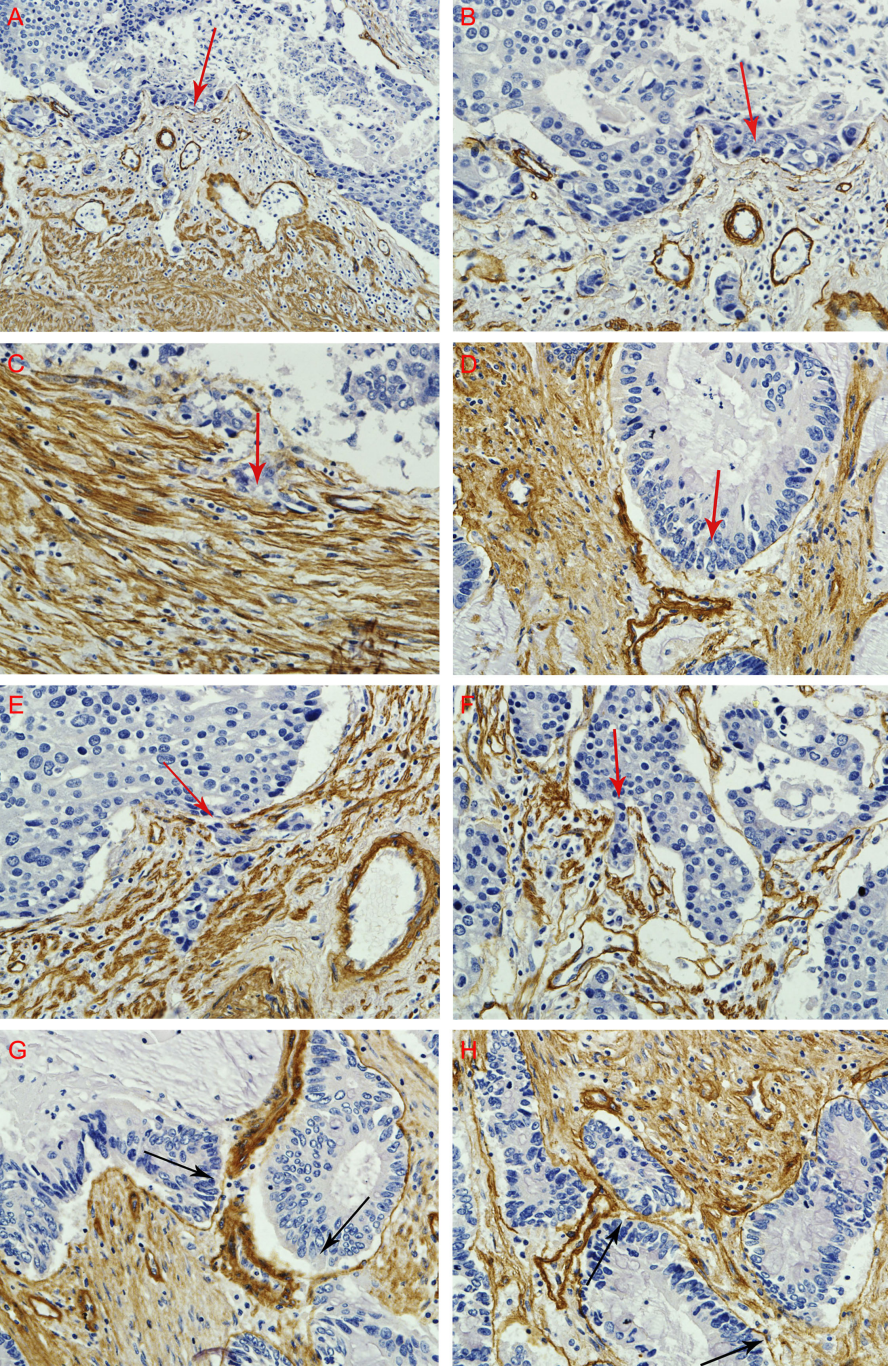
**Patterns of GC invasion**. (A, B) Washing pattern: cancer cells encroach extracllular matrix everywhere, like wave breaking the dike on the beach. (C) Ameba-like pattern: after breaking the collagen, cancer cells invade ECM along the interspace of collagen on both sides to form an Ameba-like ulcer. (D) Spindle pattern: cancer cells proliferate with polarity, and the collagen at the tumor-invasion front is hydrolyzed to overcome the ECM barrier, forming a potential invasive tunnel. (E, F) Linear pattern: cancer cells digest the ECM main along a line. (G, H) Type IV collagen was abruptly degraded at a point, several cells were migrating (G). Though type IV collagen was not broken, degradation was obvious. Magnifications: A: 200×, B-H: 400×. Red arrows present the trend of invasion. Black arrows indicate the breaking points of IV collagen by hydrolysis. All tissues were adenocarcinoma of GC.

Four typical invasion patterns were observed at the histological level. 1. Washing pattern. Cancer cells erase ECM everywhere without foci degraded matrix, like wave breaking the dike on the beach (Figure 4A &4B). 2. Ameba-like pattern. After breaking the collagen, cancer cells invade ECM along the interspace of collagen on both sides to form an Ameba-like ulcer (Figure 4C). 3. Spindle pattern. Cancer cells proliferate with polarity, and the collagen at the tumor-invasion front is hydrolyzed to overcome the ECM barrier, forming a potential invasive tunnel (Figure 4D). 4. Linear pattern. Cancer cells hydrolyze the ECM at one focal point and the invasion trace displays as a line (Figure 4E, F).

Invasion analysis observed that type IV collagen was abruptly degraded at a point, through which only a few cancer cells were crossed (Figure 4G). Invasion maybe have already occurred even though type IV collagen was not broken because the degradation became obvious (Figure 4H).

## Discussion

Invasion is the first observable step of cancer progression. Cancer invasion occurs in a particular context of tissue microenvironment which is under constant evolution largely due to the interactions of cancer cells and the surrounding stromal cells [13,14]. However, such co-evolution of cancer-microenvironment has long been under appreciated. Most studies focused on molecular level gene mutations and signal pathways in cancer cells during tumor progression, while other studies focused on TNM staging at the clinical level [15,16]. The molecular level studies focused on the "temporal evolution" of cancer molecules, while the clinical studies focused on the "spatial evolution" of cancer tissues. The underlying theory behind these studies is to focus on cancer itself. A major drawback of such study, however, is the lack of appreciation of the "temporal and spatial co-evolution of cancer and its environment", which is the real context of tumor progression [17].

It is based on such understanding that this study focused on major ingredients of tumor microenvironment, particularly the cancer invasion front, as well as cancer cells. These components included in this study were MMPs and type IV collagen, two major factors for and against cancer invasion, and TAMs which are double-edge swords facilitating or deterring cancer invasion. Moreover, tumor angiogenesis was also evaluated because provides potential routes for tumor dissemination as a result of the co-evolution of cancer microencironment and cancer cells and promoted by those components.

MMPs are major proteolytic enzymes to breakdown ECM during cancer invasion. Traditionally, extracellular proteolysis and BM breaching are two absolute requirements for cancer invasion, while type IV collagen forms physical barrier against cancer invasion [18]. High levels of proteases facilitate ECM degrading, thereby creating a path for the migration of cancer cells. As a result of this path through the ECM, the invading cancer cells could gain access to vasculature and lymphatic systems [19]. This progress would rely on invadopodia which are membrane protrusions that localize enzymes required for ECM degradation, and MMP9 would be required in the initial steps of invadopodia formation [20]. In support to this theory, this study revealed high expression of MMP9 in advanced GC tumor tissue, especially nearby the BM. Although the difference of MMP2 expression is significant in terms of the recurrence and metastatic status, the MMP9 expression was not associated with tumor stage, lymph node status, metastasis status, recurrence or not. Similar unexpected result was showed in terms of the relationship of type IV collagen and tumor progression.

Tumor microenvironment plays dynamic and different roles in different stages of cancer progression, which could partly explain these unexpected results. It has been evident that although cancer cells and some traditionally proteins account for invasion and metastasis are no different, the microenvironments at the primary tumor site, the invasive front and the metastatic site are different [21]. Although no statistically significant result was showed regarding of the relationship of type IV collagen and tumor progression, OS was significantly improved in type IV collagen positive group compared to negative group (the median OS was 25.5 months and 10.0 months, respectively, *P *= 0.044). Further more, GC patients with negative MMP9 expression displayed improved overall survival compared to patients with positive MMP9 expression (Median OS was 44.0 and 13.5 months, respectively. *P *= 0.036). Nevertheless, the roles of proteases in cancer are now known to be much broader than simply degradation of ECM during tumor invasion and metastasis. The proteolysis of ECM by MMPs may reveal cryptic matrix binding sites, MMPs can act as tumor suppressor by revealing cryptic matrix binding sites, releasing matrix-bound growth factors and activating a variety of cell surface molecules [22]. For instance, angiostatin and tumstatin are angiogenesis inhibitors generated from the NC1 domain of the 3 chain of type IV collagen [23]. Thus, we supposed that MMPs-mediated degradation of BM and ECM can act as both positive and negative regulators of tumor progression which resulted in the unexpected results predicted in the traditional view because of the change of the tumor stroma during the cancer progression.

Macrophages are versatile, plastic inflammatory cells that respond to environmental signals with polarized genetic and functional programs. The presence and significance of macrophages infiltration in developing neoplasms is now well recognized, and infiltrating macrophages play an important role in tumor cell invasion into surrounding normal tissues [24,25], including expression of growth factors, matrix proteases, promotion of angiogenesis and suppression of adaptive immunity, all of which influence the ECM and hypoxia, two non-cellular components that potently influence stromal-epithelial interactions [21,26] (Figure 1). A protumoral role of tumor-associated macrophages (TAMs) is consistent with studies from humans, wherein a high density/number of TAMs is associated with poor prognosis in different cancers (cervix, prostate, breast, bladder) [27,28]. In agreement with these results, our study also found that macrophages infiltration was correlated with serosa invasion, distant metastasis and TNM stage. The OS was longer in low macrophages density group than in high macrophages density group, although the level of significance was only marginal (*P *= 0.056). Additionally, the cumulative disease-free survival (DFS) rate was significantly higher in low macrophages density group than in high macrophages density group. We found that the interface of tumor nest and stroma is the main location of infiltrating macrophages in gastric cancer, and phagocytosis of cancer cells by macrophage, indicating the coexistence of M1 and M2 phenotypes in GC tissues.

In cancer, tumor cells require new blood vessels for sustenance, local growth and escape to distant sits through hematogenous spreading and metastasis [29]. No matter the mechanism of the invasion, angiogenesis maybe the common last step of invasion in primary tumor environment. In our study, tumor angiogenesis was studied by calculating the MVD, and the MVD was higher in patients with GC lymph node metastasis and advanced GC (*P *= 0.019 and 0.010, respectively). Interestingly, our results indicate that type IV collagen and macrophages were the negative and positive factors for tumor angiogenesis, respectively, in keeping with what we have mentioned above. In the early stage, MMPs destroy the ECM and established a potential pathway for cancer cell migration but the revealed molecule from type IV collagen inhibits the tumor angiogenesis [30]. Whereas in the advanced stage, type IV collagen was almost destroyed and no molecules that inhibit tumor angiogenesis were released, that's why MVD was higher in type IV collagen negative group than in positive group (*P *= 0.026). It has been well established that M2 type macrophages can promote the tumor angiogenesis [31], and we found that MVD was higher in high density macrophages group than in low density group (*P *= 0.040). Histomorphology analysis also indicates that the locations of infiltrating macrophages and MVD are accordant (Figure 2E and Figure 2F). One limitation of this study, however, is that it did not differentiate between M1 and M2 cells. Further work in this direction would be more informative.

The current study suggests that GC invasion is influenced by co-evolution of cancer cells and their microenvironment, and histological study on tumor tissue can directly show such interactions. Based one our observations, we analyzed invasion patterns in an attempt to characterize the invasive behaviours of GC beyond the simplistic gene mutation or overall TNM stage, whose values were limited for ignoring the interaction of cancer cells and stroma. Rather, this study focused on the micro-ecology system of cancer invasion front (Figure 1), and identified four invasive patterns, including Washing pattern, Ameba-like pattern, Spindle pattern, and Liner pattern, each representing distinctive interactions between cancer cells and their microenvironment. In the Washing pattern, successive waves of cancer cells may induce progressive conditioning of the microenvironment to facilitate cancer cells spreading along a plane rather than deep penetration. In the Ameba-like pattern, extensive tissue destruction may have occurred in the adjacent tissue even though the local tumor border appears intact. Therefore, invasive tunnels may have already developed beneath the seemly intact tumor margin. In the Spindle pattern, simultaneous coordinated polarization of cancer cells at the leading edge of tumor front may cooperate in invasion by constantly changing the local microenvironment. In linear pattern, a few coordinated "pioneering cancer cells" form deep penetrating invasion tunnels along a line, paving the way for follower cancer cells. Among these four patterns, washing pattern may correlate with best prognosis as crossing ECM barriers occurs relatively late. In contrast, Linear pattern may relate to the worst prognosis because cancer cells may have already deeply penetrated the ECM in spite of the density of the surrounding type IV collagen, and such cancer may have already become a potentially systemic disease even it is diagnosed as early stage by conventional pathology. However, the significance of invasion patterns was not fully evaluated in this study because of the limited sample size, which is the major limitation of our study. Large scale studies are needed to further develop this concept.

## Conclusions

In summary, proteolytic enzymes MMP9, MMP2 and macrophages in stroma contribute to GC progression by facilitating tumor angiogenesis. The co-evolution of tumor cells and their microenvironment results in four patterns of tumor invasion, which could be useful for new prognostic models and novel treatment strategies.

## List of abbreviations

GC: Gastric Cancer; MVD: Microvessel density; BM: Basement Membrane; MMP: Matrix Metalloproteinases; OS: Overall Survival; DFS: Disease-free Survival; TAMs: Tumor-associated Macrophages

## Competing interests

The authors declare that they have no competing interests.

## Authors' contributions

PCW selects the research topic, conducts the pathological examination, statistical analysis and writes manuscript. LXL and LX conduct the pathological examination. LY conceives the study project, organizes the whole study process, provides financial support, and finalizes the manuscript. All authors have read and approved the final manuscript.
